# Early Gestational Diabetes Mellitus: Diagnostic Strategies and Clinical Implications

**DOI:** 10.3390/medsci9040059

**Published:** 2021-09-23

**Authors:** Saptarshi Bhattacharya, Lakshmi Nagendra, Aishwarya Krishnamurthy, Om J. Lakhani, Nitin Kapoor, Bharti Kalra, Sanjay Kalra

**Affiliations:** 1 Department of Endocrinology, Max Hospital, New Delhi 110092, India; 2 Department of Endocrinology, K.S Hegde Medical Academy, Mangalore 575018, India; drlakshminagendra@gmail.com; 3 Department of Endocrinology, Max Hospital, Ghaziabad 201012, India; aishwaryakr1410@gmail.com; 4 Department of Endocrinology, Zydus Hospital, Ahmedabad 380058, India; dromlakhani@gmail.com; 5 Department of Endocrinology, Diabetes and Metabolism, Christian Medical College, Vellore 632004, India; nitin.endocrine@gmail.com; 6 Department of Obstetrics, Bharti Hospital, Karnal 132001, India; dr.kalrabharti@gmail.com; 7 Department of Endocrinology, Bharti Hospital, Karnal 132001, India; brideknl@gmail.com

**Keywords:** gestational diabetes mellitus, early diagnosis, early treatment, fasting hyperglycemia, oral glucose tolerance test, large-for-date baby

## Abstract

Preexisting diabetes mellitus (DM) should be ruled out early in pregnancy in those at risk. During screening, a significant proportion of women do not reach the threshold for overt DM but fulfill the criteria used for diagnosing conventional gestational DM (cGDM). There is no consensus on the management of pregnancies with intermediate levels of hyperglycemia thus diagnosed. We have used the term early gestational DM (eGDM) for this condition and reviewed the currently available literature. Fasting plasma glucose (FPG), oral glucose tolerance test, and glycated hemoglobin (HbA1c) are the commonly employed screening tools in early pregnancy. Observational studies suggest that early pregnancy FPG and Hba1c correlate with the risk of cGDM and adverse perinatal outcomes. However, specific cut-offs, including those proposed by the International Association of the Diabetes and Pregnancy Study Group, do not reliably predict the development of cGDM. Emerging data, though indicate that FPG ≥ 92 mg/dL (5.1 mmol/L), even in the absence of cGDM, signals the risk for perinatal complication. Elevated HbA1c, especially a level ≥ 5.9%, also correlates with the risk of cGDM and worsened outcome. HbA1c as a diagnostic test is however besieged with the usual caveats that occur in pregnancy. The studies that explored the effects of intervention present conflicting results, including a possibility of fetal malnutrition and small-for-date baby in the early treatment group. Diagnostic thresholds and glycemic targets in eGDM may differ, and large multicenter randomized controlled trials are necessary to define the appropriate strategy.

## 1. Introduction

The prevalence of diabetes mellitus (DM) has been steadily increasing [1]. It has been accompanied by a parallel rise in the occurrence of gestational diabetes mellitus (GDM) [2]. This is partly attributed to modifications in the criteria for diagnosis. However, the dominant etiology behind the surge in GDM cases is related to the increase in the prevalence of obesity and DM in the reproductive age group [2,3].

The two-step oral glucose tolerance test (OGTT) proposed by Carpenter–Coustan had long been the accepted test for GDM [4]. The International Association of the Diabetes and Pregnancy Study Group (IADPSG) in 2010, based on the findings of the “Hyperglycemia and Adverse Pregnancy Outcome” (HAPO) study, recommended a one-step criterion [5,6].

The IADPSG also advocated that pregnant women should be evaluated for overt DM early in pregnancy. This early testing strategy, subsequently endorsed by the World Health Organization (WHO) and most other societies, led to the recognition of a cohort of women in early pregnancy who manifest intermediate degrees of hyperglycemia [7]. Glycemic levels before 24 weeks gestation, falling short of the criteria for overt DM but fulfilling the requirements for conventional GDM (cGDM), are labeled as early GDM (eGDM). There is a need to understand this entity better, as it could streamline and economize screening strategies for hyperglycemia in pregnancy and offer an opportunity to intervene earlier than usual to improve maternal and fetal outcomes.

## 2. Objectives of the Review

This review analyzes the currently available evidence on eGDM. The predictive ability of fasting plasma glucose (FPG), the oral glucose tolerance test (OGTT), and glycated hemoglobin (HbA1c) in early pregnancy to detect cGDM and perinatal outcome has been studied. We also reviewed the trials analyzing the impact of lifestyle and therapeutic interventions on eGDM. The best possible strategy for the diagnosis and treatment of eGDM in the light of the currently available evidence are discussed.

## 3. Why Is This Topic Important?

Intermediate levels of hyperglycemia, or eGDM, is a commonly encountered clinical conundrum in early pregnancy. The current diagnostic strategies for eGDM are not based on adequate evidence. The criteria for the diagnosis of cGDM have been applied for convenience in early pregnancy. However, newer data indicate that intermediate levels of hyperglycemia in early pregnancy may be associated with an increased risk of adverse outcomes. Thus, an earlier diagnosis might offer a chance for timely intervention and improve pregnancy results.

The available guidelines do not address the management of eGDM due to insufficient evidence. The therapeutic approach to such cases is not clearly defined. There is a need to analyze the evidence and plan further research to formulate an appropriate testing and therapeutic strategy for eGDM.

## 4. Definition of Terms

**Gestational diabetes mellitus:** GDM refers to hyperglycemia diagnosed between 24 and 28 weeks of pregnancy by the standard criteria (conventionally IADPSG or two-step OGTT) but falling short of the levels for overt diabetes [4,6,8]. The term cGDM is used for this condition in our review.

**Overt Diabetes** or **Diabetes in Pregnancy:** This refers to hyperglycemia diagnosed during pregnancy, satisfying the standard criteria (ADA or WHO) for DM in nonpregnant individuals [9,10]. We have used the term “overt diabetes” in this review.

**Early gestational diabetes mellitus:** eGDM refers to intermediate degrees of hyperglycemia detected before 24 weeks of pregnancy that fulfill the criteria for cGDM but fall short of the threshold for overt diabetes [6,8,9].

## 5. Literature Search Strategy

We conducted a PubMed search to identify articles published until July 2021 on GDM or hyperglycemia diagnosed during the first and second trimesters of pregnancy using the following search strategy. The terms “gestational diabetes mellitus”, “diabetes in pregnancy”, “hyperglycemia in pregnancy”, “glucose intolerance in pregnancy”, and “fasting hyperglycemia in pregnancy” were searched in combination with “early diagnosis”, “early screening”, “early treatment”, “early pregnancy”, “booking visit”, “first prenatal visit”, “first antenatal visit”, “first trimester”, and “second trimester”. Relevant articles were also identified through Google Scholar. The references of these articles were scanned and reviewed if found suitable.

## 6. Current Guidelines for Detecting Gestational Diabetes Mellitus at 24–28 Weeks

Table 1 summarizes the current guidelines recommended for the diagnosis of cGDM. The two most commonly used ones are the two-step OGTT using Carpenter–Coustan criteria and the one-step OGTT with IADPSG cut-offs [4,6]. The IADPSG in 2010, based on the findings of the HAPO study, suggested the one-step criterion [4,5,6]. However, the American College of Obstetricians and Gynecologists (ACOG) continues to recommend the traditional two-step approach [8].

Both the Carpenter–Coustan and the IADPSG criteria have limitations. The Carpenter–Coustan criteria were based on the maternal risk of developing type 2 DM (T2DM) in the future and not construed on feto-maternal effects [4,11]. The recommendations from IADPSG attempted to redefine the diagnostic criteria of cGDM in terms of adverse pregnancy outcomes. The OGTT cut-offs for the diagnosis of cGDM were established when the odds ratio to develop an adverse event reached 1.75-fold in the HAPO study as compared to the mean values of the study population [5]. The IADPSG criteria offer a simplified one-step method and utilize the same 75 g 2 h OGTT protocol as universally accepted for testing outside of pregnancy. The primary drawback remains the risk of overdiagnosis. Adopting this criterion has led to a one- to three-fold increase in cGDM in different studies [12].

Trials analyzing treatment outcomes of cGDM diagnosed by IADPSG criteria versus those diagnosed by conventional approaches have not convincingly demonstrated the benefits of one over the other. A recently published large pragmatic randomized trial showed no difference in perinatal and maternal outcomes despite more cases being diagnosed with the one-step criterion [13]. It is often argued that the additional medical and financial implications of the aggressive one-step approach might not be justified [14]. Nevertheless, the IADPSG criterion has been adopted by the majority of health organizations [7,15,16,17,18,19]. In the absence of definitive evidence in favor of either, the strategy to adopt a criterion must consider the regional prevalence of cGDM, the institutional practice, the available infrastructure, and the cost effectiveness.

**Table 1:** Diagnostic criteria for GDM at 24–28 weeks.

Organization/Society	Criteria	Method	Tests	Interpretation
American Diabetes Association (2021) [18] Endocrine Society (2013) * [19] World Health Organization (2013) and International Federation of Gynecology and Obstetrics (2015) ** [7,16] Australasian Diabetes in Pregnancy Society (2014) [17]	IADPSG	One-step OGTT with 75 g glucose	FPG 1 h PG 2 h PG	Diagnosis of GDM is made if one of the following criteria are met FPG: ≥92 mg/dL (5.1 mmol/L) 1 h: ≥180 mg/dL (10.0 mmol/L) 2 h: ≥153 mg/dL (8.5 mmol/L)
American College of Obstetricians and Gynecologists (2018) [8]	Carpenter–Coustan/ National Diabetes data group	Step 1: non-fasting 50 g glucose screen Step 2: (3 h 100 g OGTT)	1 h PG (50 g) FPG 1 h PG 2 h PG 3 h PG	Proceed to 100 g OGTT if 1 h PG exceeds institutional thresholds (cut-off values of 130 mg/dL (7.2 mmol/L), 135 mg/dL (7.5 mmol/L), or 140 mg/dL (7.8 mmol/L) based on community prevalence rates of GDM) A diagnosis of GDM requires that two or more thresholds be met or exceeded by one of the following criteria **Carpenter–Coustan Criteria** FPG: ≥95 mg/dL (5.3 mmol/L) 1 h: ≥180 mg/dL (10.0 mmol/L) 2 h: ≥155 mg/dL (8.6 mmol/L) 3 h: ≥140 mg/dL (7.8 mmol/L) **National Diabetes Data Group** FPG: ≥105 mg/dL (5.8 mmol/L) 1 h: ≥190 mg/dL (10.6 mmol/L) 2 h: ≥165 mg/dL (9.2 mmol/L) 3 h: ≥145 mg/dL (8.0 mmol/L)
National Institute for Health and Care Excellence (2015) [15]		One-step 75 g OGTT	FPG 2 h PG	Diagnosis of GDM is made if one of the following criteria are met FPG ≥100 mg/dL (5.6 mmol/L) 2 h: ≥140 mg/dL (7.8 mmol/L)

* Diagnosis of overt diabetes is made if one of the following criteria are met: FPG: ≥126 mg/dL (7 mmol/L); 2 h post 75 g OGTT: ≥200 mg/dL (11.1 mmol/L). ** Diagnosis of Overt diabetes is made if one or more of the following are met: FPG ≥ 126 mg/dL (7 mmol/L); 2 h post 75 g OGTT ≥ 200 mg/dL (11.1 mmol/L); RBS ≥ 200 mg/dL (11.1 mmol/L) in the presence of diabetes symptoms. GDM—gestational diabetes mellitus, OGTT—oral glucose tolerance test, PG—plasma glucose, FPG—fasting PG, IADPSG—International Association of the Diabetes and Pregnancy Study Group, GCT—glucose challenge test.

## 7. Current Recommendations for Detecting Hyperglycemia in Early Pregnancy

Most associations recognize the importance of screening for hyperglycemia early in pregnancy [6,7,8,15,16,17,20]. With the increasing prevalence of undiagnosed DM in women of the reproductive age group, the primary purpose of this strategy is to detect preexisting hyperglycemia [21,22]. The compelling need to rule out overt DM in early pregnancy arises from the risk of congenital malformation associated with it [23,24]. There is a lack of consensus regarding which group of women should be screened. Most guidelines recommend testing for high-risk groups, as there is insufficient evidence to suggest universal screening at present. The accepted criteria for diagnosing overt DM are the same as those of outside pregnancy [20,25].

The importance of screening early to detect overt DM was strongly proposed by IADPSG. The group suggested any one of FPG, HbA1c, or random plasma glucose (RPG) (with subsequent confirmation) as a screening tool [6]. The WHO in 2013, and the International Federation of Gynecology and Obstetrics (FIGO) in 2015, also endorsed the necessity for early screening [7,16]. They recommended the OGTT (with the IADPSG cut-offs) to screen for DM in early pregnancy [6]. The Australasian Diabetes in Pregnancy Society (ADIPS) advocated using IADPSG criteria but suggested a risk-based approach [17]. The ADA and the ACOG proposed risk-based screening in early pregnancy but have not clarified the specific methodology because of inadequate evidence to endorse any particular strategy [8,20]. The National Institute for Health and Care Excellence (NICE), the United Kingdom, advises a 75 g OGTT with different cut-offs (FPG ≥ 100 mg/dL (5.6 mmol/L) or 2 h PG ≥ 140 mg/dL (7.8 mmol/L) for women at high risk. Table 2 summarizes the recommendations for screening for hyperglycemia in early pregnancy by various societies.

These criteria were derived from studies in women after 24 weeks of pregnancy. There is currently inadequate evidence that validates their usage earlier. Although the management strategy for overt DM in early pregnancy is standardized, the management of women with intermediate levels of hyperglycemia before 24 weeks is not defined [26,27].

## 8. Fasting Plasma Glucose for Diagnosis of eGDM

The IADPSG recommendations published in 2010, suggested the measurement of FPG to rule out preexisting hyperglycemia in early pregnancy. The committee also proposed that FPG ≥ 92 mg/dL (5.1 mmol/L) but <126 mg/dL (7.0 mmol/L) in early pregnancy should be classified as GDM [6]. The suggestion was not backed by evidence, and the committee members later withdrew the recommendation [28]. Though FPG is accepted as a simple and accurate screening tool to rule out overt DM, its usefulness in diagnosing eGDM is debatable. This section discusses the evidence related to FPG in early pregnancy as a screening tool for eGDM.

### 8.1. Changes in Fasting Plasma Glucose during Pregnancy

The FPG level drops marginally in the early half of pregnancy [29,30,31]. A study comprising 361 pregnant women observed that the maximum fall was 2 mg/dL (0.1 mmol/L) and occurred from six to ten weeks [29]. Two large retrospective studies from China reported that FPG values decreased until 16–19 weeks and stabilized after that [30,31]. In another cohort of 7946 women from a single center in Israel, the median FPG before conception was 81 mg/dL (4.5 mmol/L). The median FPG in the same group after conceiving was 80 mg/dL (4.4 mmol/L) at 4–9 weeks, decreased to 78 mg/dL (4.3 mmol/L) at 10–14 weeks, remained at 77 mg/dL (4.27 mmol/L) between 15 and 29 weeks, and showed a small drop to 76 mg/dL (4.22 mmol/L) in the last 10 weeks [32].

### 8.2. Fasting Hyperglycemia and Risk of cGDM in Observational Studies

Several observational studies have analyzed whether intermediate elevations in the first-trimester FPG correlates with a higher risk of cGDM. A positive correlation has been demonstrated in most of these reports [31,33,34,35,36,37,38,39,40,41]. A few early studies, however, did not show this association [42,43,44]. The current evidence suggests that first-trimester FPG and the risk of cGDM have a linear relationship and that higher values strongly correlate with an increased risk. Additional parameters that increased the predictive accuracy of FPG include fasting plasma insulin levels [35,37], body mass index (BMI) [31,34,39,45], high-sensitivity C-reactive protein (hs-CRP) [38], and serum triglyceride [37]. Table 3 summarizes the findings of the recent observational studies.

### 8.3. Fasting Hyperglycemia and Pregnancy Outcome in Observational Studies

One of the early studies that suggested a link between elevated early pregnancy FPG and worsened fetal outcome was published by Riskin-Mashiah et al. in 2009. The investigators reported an increased risk of large-for-gestational age (LGA) babies, macrosomia, and caesarean section (CS) at higher values of FPG. Of note, the study excluded women with an FPG of more than 105 mg/dL (5.8 mmol/L) [33]. Several other studies have shown an association between LGA and macrosomia with elevation in first-trimester FPG [36,41,46,47,48,49]. A higher FPG was also associated with pregnancy-induced hypertension (PIH) [36,46,48], prematurity and preterm birth [46,48], non-evolutive pregnancies and fetal death [50], CS and assisted vaginal delivery [41], and neonatal intensive care unit (NICU) admission [51]. An interesting observation was the higher risk of fetal complications arising from elevated FPG levels irrespective of subsequent GDM [48,51]. In a large retrospective analysis of 22,398 singleton pregnancies from China, women with medium FPG (92–100.8 mg/dL (5.1 mmol/L–5.6 mmol/L)) showed an increased risk of PIH and macrosomia. Higher values of FPG (100.8—126 mg/dL (5.6 mmol/L–7.0 mmol/L)) correlated with PIH, macrosomia, LGA, and preterm birth despite a normal OGTT between 24 and 28 weeks [48].

### 8.4. Randomized Controlled Trials on the Predictive Ability of Fasting Hyperglycemia to Detect GDM

Very few RCTs have tried to assess the ability of the different first-trimester tests to predict GDM. A randomized control trial (RCT) by Yeral et al. with 486 women compared the effectiveness of early pregnancy screening methods to detect cGDM. Intermediate values of FPG (92 to 125 mg/dL (5.1 to 6.9 mmol/L)) in the first trimester had poor specificity (77.37%) and positive predictive value (PPV) (20.33%) to identify cGDM. The negative predictive value (NPV) of FPG was 92.29%. Among FPG (5.1%), the two-step method (6.0%), and the one-step IADPSG method (11.3%), the latter most reliably detected the future risk of cGDM. The investigators concluded that until definitive evidence is available, the OGTT as per IADPSG criteria could be the preferred test in the first trimester [52].

### 8.5. Fasting Plasma Glucose and Postpartum Glucose Homeostasis

The literature on the correlation between early pregnancy fasting hyperglycemia and postpartum altered glucose homeostasis is sparse. A retrospective study of 4608 women from the Portuguese National Registry of GDM revealed that the area under the receiver operating characteristic (ROC) curves (AUC) for the association with postpartum T2DM was 0.85 (0.80–0.90) for first-trimester FPG and 0.85 (0.80–0.91) for HbA1c. An FPG cut-off of 99 mg/dL (5.5 mmol/L) showed 77.4% sensitivity, 74.3% specificity, and a PPV of 4.8% and an NPV of 99.5% for the detection of postpartum T2DM. A first-trimester HbA1c of 5.4% had a sensitivity of 79.0%, a specificity of 80.1%, a PPV of 5.7%, and an NPV of 99.6%. The investigators concluded that first-trimester FPG < 99 mg/dL (5.5 mmol/L) and HbA1c < 5.4% could be used as cut-offs to rule out the possibility of postpartum T2DM [53]. Cosma et al. did not report any difference in postpartum hyperglycemia between 192 women with eGDM (elevated FPG as per IADPSG criteria) and 81 women with cGDM diagnosed as per the one-step criterion. However, the eGDM group had less incidence of preterm labor, more induced deliveries, and reduced fetal problems [54]. More studies are required to analyze the association between postpartum dysglycemia and eGDM.

**Table 3:** Major observational studies (from 2009) analyzing the association between early pregnancy FPG to development of subsequent GDM and perinatal outcome.

Author (Year of Publication/Country)	Study Type	First Trimester and 24–28 Weeks Screening Methods	Number of Participants	Correlation to Development of GDM at 24–28 Weeks	Clinical Outcome/Comments
Riskin-Mashiah(2009/Israel) [33]	Retrospective study	FPG at first trimester 2-step OGTT (CC criteria) at 24–28 weeks with 140 mg/d (7.7 mmol/L) l cut-off for GCT ^a^	N = 6129 GDM—173	Frequency of GDM increased from 1.0% in the lowest glucose category to 11.7% in the highest (adjusted OR 11.92 (95% CI 5.39–26.37))	Frequency of LGA neonates and/or macrosomia increased from 7.9 to 19.4% (2.82 (1.67–4.76)). Primary CS rate increased from 12.7 to 20.0% (1.94 (1.11–3.41)) FPG > 105 mg/dL (5.8 mmol/L) excluded from the study.
Riskin-Mashiah(2010/Israel) [34]	Retrospective study	FPG at first trimester	N = 4876 GDM—135	**FPG cut-off—95 mg/dL (5.2 mmol/L)** Sensitivity 15.6% Specificity 95.9% PPV 9.7% NPV 97.6% AUC—0.72 ± 0.023	1.5-fold increase in the risk of GDM with each 5 mg/dL (0.2 mmol/L) increase in FPG or 3.5 kg/m^2^ increase in BMI **FPG < 80 mg/dL (4.4 mmol/L)** has an NPV of 98.6% for GDM FPG > 105 mg/dL (5.8 mmol/L) excluded from the study
		2-step OGTT (ACOG) at 24–28 weeks with 140 mg/dL (7.7 mmol/L) cut-off for GCT ^a^	FPG > 95 mg/dL (5.2 mmol/L)—216 FPG > 90 mg/dL (5 mmol/L)—531		
Yachi (2011/Japan) [35]	Prospective study	FPG and fasting insulin levels prior to week 13. GCT followed by GTT	N—509 Positive GCT—106 GDM—8	**FPG cut-off ≥ 65.88 mg/dL (3.66 mmol/L)** for detecting positive GCT Sensitivity 86% Specificity—29% AUC—0.588	First-trimester fasting plasma insulin levels improve predictive ability of FPG for subsequent GCT positivity GDM diagnosed as per Japanese guidelines [55]
Kayemba-Kay’s (2012/England)[36]	Prospective study	FPG at first antenatal visit. FPG at 28 weeks. OGT with 75 g glucose, 2 hr PG > 160 mg/dL (8.8 mmol/L)—GDM	N—1480 GDM—18	FPG 84.6–95 mg/dL (4.7–5.3 mmol/L) OR for GDM 0.65 (0.14–3.03) FPG 97.2–108 mg/dL (5.4–6.0 mmol/L) OR for GDM 1.87 (0.50–6.99) FPG 109.8–122.4 mg/dL (6.1–6.8 mmol/L) OR for GDM 1.77 (0.22–14.23) FPG > 122.4 mg/dL ( 6.8 mmol/L) OR for GDM 4.62 (1.39–15.35)	**FPG > 109.8 mg/dL (6.1 mmol/L)** associated with increased risk of PIH **FPG > 122.4 mg/dL (6.8 mmol/L)** higher likelihood of macrosomic baby (OR 3.1 (95% CI: 1.21–8.0))
Corrado (2012/Italy) [56]	Retrospective study	FPG at first trimester OGTT (IADPSG) at 24–28 weeks	N—738 GDM—88 First-trimester FPG ≥ 92 mg/dL (5.1 mmol/L)—53	**FPG > ≥ 92 mg/dL (5.1 mmol/L)–** GDM—45.3% [24] FPR—4.4% FNR—9.8% OR—8.0 (95% CI: 4.4–14.6),	OR for GDM adjusted for pre-pregancy BMI and maternal age—7.1% (95% CI: 3.8–13.1)
Zhu (2013/China) [30]	Retrospective study	FPG at first prenatal visit	N—17186	FPG level at first prenatal visit strongly correlated with risk of GDM at 24–28 weeks (*p* < 0.001). Incidences of GDM were 37.0, 52.7, and 66.2%, respectively, for FPG between 91.8 and 100.62 mg/dL, 100.8 and 109.62, 109.8 and 125.8 mg/dL (5.10 and 5.59, 5.60 and 6.09, and 6.10–6.99 mmol/L)	AUC -0.654 (95% CI 0.643–0.665) **FPG cut point of 100.8 mg/dL (5.60 mmol/L)**, specificity—0.99 Cut point of 109.8 mg/dL (6.10 mmol/L), specificity—1
		OGTT (IADPSG) at 24–28 weeks	GDM—3002		
Shuang (2014/China) [37]	Retrospective study	FPG in early pregnancy	N—427	Elevated FPG in early pregnancy increased risk for GDM (OR:4.03, 95%CI: 1.62–10.02)	FPG, triglyceride, fasting insulin level during early pregnancy risk factors for GDM
		1-step 75 g OGTT at 24–28 weeks	GDM—74		
Ozgu-Erdinc (2014/Turkey) [38]	Prospective study	FPG at week 11 to 13	N—439	**At FPG cut-off of 90 mg/dL ( 5 mmol/L)** Sensitivity 55.1 (40.3–69.1) Specificity 71.0 (66.2–75.4) PPV 19.3 (13.3–27.0) NPV 92.6 (88.9–95.2) OR 3.0 (1.7–5.5) AUC 0.609 (*p* = 0.034)	FPG and hs-CRP in first trimester correlated with development of GDM. FPG had a better sensitivity; hs-CRP had better specificity.
		2-step OGTT (CC criteria) at 24–28 weeks	GDM—49		
Fahami (2015/Iran) [44]	Prospective study	FPG at 8–13 weeks	N—88	Mean FPG who had GCT < 140 mg/dL (7.7 mmol/L)— 80.48 ± 8.21 mg/dL (4.4 ± 0.45 mmol/L) Mean FPG with those haiving GCT ≥ 140 mg/dL (7.7 mmol/L)— 82.8 ± 9.29 mg/dL (4.6–0.5 mmol/L) **At FPG cut-off 79.5 mg/dL (4.4 mmol/L)** Sensitivity 60% Specificity 45.2% PPV 18.4% NPV 84.6% AUC 0.573	FPG < 95 mg/dL included in the study FPG had limited usefulness in predicting positive GCT Complimentary fasting plasma insulin—no additional value
		GCT (cut-off 140 mg/dL) at 24–28 weeks	GCT > 140 mg/dL—15		
Hao (2017/China) [39]	Retrospective study	FPG at 8–9 weeks. OGTT (IADPSG) at 24–28 weeks	N—820 GDM—167	**At FPG cut-off point of 83 mg/dL (4.6 mmol/L)** Sensitivity 53.89% (46.33–61.45) Specificity 70.90% (67.42–74.39) PPV 32.14% (26.67–37.61) NPV 85.74% (82.79–88.69) AUC 0.666 (0.619–0.714)	FPG and BMI combined enhanced predictability for GDM (OR, 3.861; 95% CI: 2.701–5.520) FPG ≥ 83 mg/dL (4.6 mmol/L) in 300; 93 (31.0%) had GDM, 207 (69.0%) did not
Sesmilo (2017/Spain) [46]	Retrospective study	Second-trimester FPG	N—5203	Categories of second-trimester FPG—1 < 75, 2: 75–79, 3: 80–84, 4: 85–89, 5: 90–94, 6: 95–99 and 7: 100–124 mg/dL (1 < 4.16, 2: 4.16–4.38, 3: 4.44–4.66, 4: 4.72–4.94, 5: 5–5.22, 6: 5.27–5.5 and 7: 5.55–6.88 mmol/L)—Risk of GDM not specified	Second-trimester FPG associated with LGA (*p* < 0.001), GHD (*p* = 0.004) and prematurity both <37 and <34 weeks of gestation (*p* = 0.001 and *p* = 0.004) and inversely related to SGA, not related to C-section, APGAR score, or macrosomia
Wei (2019/China) [31]	Retrospective study	FPG test at first prenatal visit (before 24 weeks) OGTT (IADPSG) at 24–28 weeks	N—34087 GDM—6806	FPG range—% developing GDM 92–100.62 mg/dL ( 5.10–5.59 mmol/L)—35.4% (1759/4965) 101–109.62 mg/dL (5.60–6.09 mmol/L)—56.6% (422/745) 110–125.8 mg/dL (6.10–6.99 mmol/L)—52.0% (90/173)	The predictive ability of FPG to detect subsequent GDM increased with BMI
Falcone (2019/Italy) cohort study [40]	Prospective study	Before 15 + 6 weeks FPG, fasting insulin and C-peptide, HbA1c	N—574 GDM—103	NGT—mean FPG—80.4 ± 5.6 mg/dL (4.5 ± 0.3 mmol/L) GDM— mean FPG—85.1 ± 7.4 mg/dL (4.7 ± 0.4 mmol/L) *p* Value < 0.001 AUC 0.681 (0.623–0.74) OR for GDM—1.13 (1.09–1.18) OR for GDM-PT—1.20 (1.14–1.27)	FPG showed moderate-to-fair accuracy in predicting GDM later as per AUC Better accuracy to detect GDM in need for PT (AUC—0.798)
Li (2019/China) [41]	Retrospective study	FPG at 9–13^+6^ weeks OGTT (IADPSG) at 24–28 weeks	N—2112 GDM—224	**At FPG cut-off point of 81 mg/dL (4.5 mmol/L)** Sensitivity 64.29% (57.6–70.6) Specificity 56.45% (54.2–58.7) PPV 14.9% NPV 93% AUC 0.63 (0.61–0.65)	GDM at 24–28 weeks, LGA and assisted vaginal delivery/CS was significantly higher in upper vs. lower quartile of FPG
Ozgu-Erdinc (2019/Turkey) [57]	Retrospective study	FPG before 14 weeks of pregnancy 2-step OGTT (C–C criteria) at 24–28 weeks	N—2605 GDM—245	**At FPG cut-off point of 92 mg/dL** Sensitivity 52.46% (46.00–58.84) Specificity 81.16% (79.51–82.71) PPV 22.38% (19.07–26.06) NPV 94.28 (93.16–95.23)	Best accuracy according to Youden’s index with the cut-off value of **87.5 mg/dL** **(4.8 mmol/L)** with a sensitivity 70.1% (95% CI 63.8–75.7) and specificity 66.2% (95% CI 64.2–68.1) Sensitivity and specificity of 23.6 and 94.5% for a cut-off value of **99.5 mg/dL (5.5 mmol/L)**
López Del Val (2019/Spain) [49]	Retrospective	FPG at first trimester 2-step OGTT (C–C criteria) at 24–28 weeks	N—1425	**FPG ≥ 92 mg/dL** Sensitivity 46.4% Specificity 88.8%	Higher newborn weight and higher rate of macrosomia (6.9% versus 3.5%; *p* < 0.05). The association persisted after excluding patients diagnosed with and treated for GDM.
Sesmilo (2020/Spain) [47]	Retrospective study	FPG at first trimester GDM—by NDDG criteria	N—6845 GDM—695	First-trimester FPG quartiles ≤ 78, 79–83, 84–87 and ≥ 88 mg/dL GDM risk—7, 8, 10.2 and 16% in each quartile (*p* < 0.001) Risk of second-trimester glucose > 92 mg/dL—2.6, 3.8, 6.3 and 11.4% in each quartile (*p* < 0.001)	First-trimester FPG associated with LGA (8.2, 9.3, 10 and 11.7% in each quartile, *p* = 0.011) but not with other obstetrical outcomes
Kansu-Celik (2021/Turkey) [58]	Retrospective study	FPG and HbA1c at first trimester 2-step OGTT (CC criteria) at 24–28 weeks	N—608 GDM—69	Median HbA1c and FPG concentrations significantly higher in GDM (n = 69) (5.31 ± 0.58% versus 5.01 ± 0.45%, *p* < 0.001 and 89.74 ± 8.71% versus 84.09 ± 9.16%, *p* < 0.001, respectively)	Cut-off value with highest Youden index—HbA1c levels above 5.6% with a sensitivity of 34.78%, specificity of 89.8%, with a diagnostic accuracy of 83.55% **FPG levels above 86.85 mg/dL (4.8 mmol/L)** have a sensitivity of 69.57%, specificity of 61.78%, with a diagnostic accuracy of 62.66%
Benhalima (2021/Belgium) [51]	Prospective cohort study	FPG between 6 and 14 weeks	N—2006	Sensitivity of FPG between 92 and 99 mg/dL (5.1 to 5.5 mmol/L to detect GDM—37.2% (29/78). 200/1760 (10.9%) with FPG < 92 mg/dL ( 5.1 mmol/L) developed GDM	Significantly higher rate of NICU admissions in the high fasting-NGT group compared to the low fasting-NGT group High fasting-NGT group had higher BMI, higher insulin resistance with more impaired insulin secretion and higher FPG and 30 min glucose levels on OGTT
		FPG ≥ 100.8 mg/dL (5.6 mmol/L) excluded	FPG between 92 and 99 mg/dL (5.1 to 5.5 mmol/L)—78		
		2 h 75 gm OGTT at 24–28 weeks—divided into GDM and NGT groups	GDM—229		
Babaniamansour (2021/Iran) [59]	Cross-sectional study	FPG at first prenatal visit (FPG)	N—952	**FPG cut-offs 85 and 90 mg/dL (4.7 and 5 mmol/L)** at first prenatal visit and at 24–28 weeks had excellent specificity and PPV diagnosing GDM	**FPG cut-offs 75 and 80 mg/dL (4.16 and 4.44 mmol/L)** at first prenatal visit and at 24–28 weeks can rule out GDM with high sensitivity and NPV
		OGTT at 24–28 weeks	GDM—12.7%		
Saraiva (2021/Portugal) [50]	Retrospective study	Group 1—FPG before 12 weeks ≥ 92 and <126 mg/dL (≥5.1 and <7 mmol/L)	Total GDM– 18518	No significant difference in maternal morbidity parameters in two groups	Non-evolutive pregnancies (1.1 vs. 0.1%, *p* < 0.001) and fetal death (0.6 vs. 0.2%, *p* < 0.001) more common in group 1 Congenital malformations similar in the two groups (3.2 vs. 2.8%, *p* = 0.155)
		Group 2—OGTT (IADPSG) after 12 weeks	Group 1—34.4%		
Rashidi (2021/Iran) [60]	Prospective cohort study	FPG at first visit in first trimester	N—1270 GDM—454	**At FPG cut-off of 85.5 mg/dL (4.75 mmol/L)** Sensitivity 71% Specificity 69% AUC—0.80 (95% CI: 0.76–83)	AUC in combination with BMI ≥ 25 kg/m^2^—0.85 (CI, 0.82–0.88) AUC in combination with family history of DM—0.84 (CI, 0.79–0.89)
Wang (2021/China) [48]	Retrospective study	First-trimester FPG	N—22398	Women divided into 3 groups on basis of first-trimester FPG: low < 92 mg/d (5.1 mmol/L); medium ≥ 92 < 100.8 mg/dL (5.1 to < 5.6 mmol/L) high ≥ 100.8 mg/dL to <126 mg/dL (5.6 to < 7.0 mmol/L) Abnormal OGTT in 3 groups Low—17.2% Medium—32% High—53.8% *p* value < 0.001	Medium FPG + normal OGTT—same risk of PIH and macrosomia as abnormal OGTT High FPG + normal OGTT—same risk of PIH, macrosomia, LGA, and preterm birth as abnormal OGTT Moderate or high FPG and BMI ≥ 24 kg/m^2^ even with normal OGTT higher risk of PIH, macrosomia and LGA
		OGTT (IADPSG) at 24–28 weeks	Abnormal OGTT at 24–28 weeks—4620 GDM—4446DM—174		

^a^ FPG levels were analyzed in seven categories: <75, 75–79, 80–84, 85–89, 90–94, 95–99, and 100–105 mg/dL (<4.16, 4.16–4.38, 4.44–4.66, 4.72–4.94, 5–5.22, 5.27–5.5, and 5.55–6.88 mmol/L). FPG—fasting plasma glucose, OGTT—oral glucose tolerance test, CC—Carpenter–Coustan, GCT—glucose challenge test, GDM—gestational diabetes mellitus, OR—odds ratio, LGA—large for gestational age, CS—cesarean section, PPV—positive predictive value, NPV—negative predictive value, AUC—area under curve, 2 h PG—2 h post glucose, PIH—pregnancy-induced hypertension, IADPSG—International Association of the Diabetes and Pregnancy Study Group, BMI—body mass index, hs-CRP—high-sensitivity C-reactive protein, HbA1c—glycated hemoglobin, NGT—normal glucose tolerance, NDDG—National Diabetes Data Group, GHD—gestational hypertensive disease, SGA—small for gestational age, PT—pharmacotherapy, NGT—normal glucose tolerance, NICU—neonatal intensive care unit.

### 8.6. Fasting Plasma Glucose Cut-offs for Diagnosis of eGDM

Most studies demonstrate that first-trimester FPG correlates with the risk of cGDM and perinatal outcomes, such as LGA (summarized in Table 1). Riskin-Mashiah et al. retrospectively analyzed FPG values divided into seven groups starting from <75 to 105 mg/dL (4.16 to 5.8 mmol/L) and observed that the risk of LGA neonates, macrosomia, primary CS, and cGDM increased progressively with each 5 mg/dL (0.2 mmol/L) rise in FPG [33,34]. Sesmilo et al. also demonstrated that with each quartile rise in FPG, the risk of cGDM and LGA proportionately increased [47]. In a large retrospective population-based study, 52% of women with FPG between 110 and 125.8 mg/dL (6.1–6.99 mmol/L) developed cGDM compared to 35.4% in those between 92 and 100.62 mg/dL (5.10–5.59 mmol/L) [31].

Though higher first-trimester FPG values signal a tendency toward a worse pregnancy outcome, a specific threshold to define eGDM has not been established. The FPG cut-off of 92 mg/dL (5.1 mmol/) in early pregnancy demonstrates poor specificity and sensitivity in predicting cGDM [31,51,57]. Observational studies have also explored first-trimester FPG levels of 66 mg/dL (3.66 mmol/L) [35], 79.5 mg/dL (4.4 mmol/L) [44], 81 mg/dL (4.5 mmol/L) [41], 83 mg/dL (4.6 mmol/L) [39], 85.5 mg/dL (4.75 mmol/L) [60], 87 mg/dL (4.83 mmol/L) [58], 87.5 mg/dL (4.86 mmol/L) [57], 90 mg/dL (5 mmol/L) [38], 95 mg/dL (5.27 mmol/L) [34], 101 mg/dL (5.6 mmol/L), and 110 mg/dL (6.1 mmol/L) [30], with sensitivity ranging from 15 to 71% and specificity varying between 29 and 100%. A low FPG value in early pregnancy (<80 mg/dL (4.4 mmol/L)) has a strong NPV for cGDM [34,39,40,41,44]. In the study by Corrado et al., FPG ≥ 92 mg/dL (5.1 mmol/L) although not diagnostic, was predictive of cGDM [56].

First-trimester FPG ≥ 92 mg/dL (5.1 mmol/L), as well as 100.8 mg/dL (5.6 mmol/L), indicate a higher risk of macrosomia, LGA, non-evolutive pregnancies, fetal death, PIH, and NICU admissions [36,48,49,50,51]. In a recently published large retrospective study analyzing 22,398 singleton pregnancies, FPG ≥ 92 mg/dL (5.1 mmol/L) and a normal OGTT between 24 and 28 weeks had a similar pregnancy outcome as cGDM [48]. These findings indicate that elevated FPG in early pregnancy might pose an additional perinatal risk irrespective of the development of cGDM. Further consideration could be the inclusion of BMI as another predictor of worsened perinatal events [61].

The limitations of the current literature are predominantly retrospective nature of the studies, the exclusion of pregnancies with higher FPG values in many analyses, and the lack of consistency in methods to diagnose cGDM. In addition, most of the investigators considered cGDM as the primary end-point rather than assessing pregnancy outcomes.

### 8.7. Summary of FPG as a Diagnostic Test for eGDM

While FPG is accepted as a simple and accurate screening tool to rule out overt DM, its usefulness in diagnosing eGDM is debatable. There is no consensus on a single FPG threshold to diagnose eGDM. Studies attempting to define an FPG cut-off for eGDM have explored various values ranging between 66 and 110 mg/dL (3.6–6.1 mmol/L). Of note, the most widely used cut-off value of 92 mg/dL (5.1 mmol/L) in observational studies demonstrated a poor sensitivity and specificity for detecting cGDM. There is, however, evidence to support an association between first-trimester fasting hyperglycemia (≥92 to 125 mg/dL (5.1 to 6.9 mmol/L)) and perinatal outcomes, even in the absence of the development of cGDM. First-trimester FPG ≥ 92 mg/dL (5.1 mmol/L), especially in women with higher BMI (≥24–25 kg/m^2^), signals a higher risk for LGA neonates and worsened perinatal end-points. Well-designed trials to define a diagnostic FPG threshold that can indicate the risk of adverse maternal and fetal outcomes are necessary.

## 9. Oral Glucose Tolerance Test for Diagnosis of eGDM

The one-step (with IADPSG cut-offs) and two-step OGTTs (usually with Carpenter–Coustan criteria) are the commonly employed tests to diagnose eGDM [4,6]. Australia (IADPSG), the United Kingdom (NICE guidelines), and some other countries recommend the one-step OGTT in the first trimester in high-risk groups [15,17]. However, there is no consensus on whether the criteria used between 24 and 28 weeks can be applied earlier.

### 9.1. OGTT Changes during Pregnancy

Physiological changes in pregnancy trigger a reduction in insulin sensitivity by 12–14 weeks. A further decline occurs by the end of the second trimester to finally reach the last trimester level of around 40–60% lower than the non-gravid state [62,63]. FPG falls marginally during the first trimester, but there is minimal change in glucose tolerance during the first half of pregnancy [29,30,31]. Continuous glucose monitoring system profiling in normoglycemic pregnancies demonstrated that even though FPG values are unchanged, the postprandial glucose levels increased around the 16th week and stay elevated till the 36th week. The levels normalized in the postpartum phase [64]. Early studies where serial 3 h OGTTs with 100 gm glucose were performed demonstrated that the 1 h and 2 h post-glucose values increased with each trimester, and even the 3 h value was elevated in the third trimester [65]. A recently published longitudinal study of 102 pregnancies at risk of GDM confirmed that the mean glucose during the OGTT increased between the first and the late second trimester (β = 8.1 mg/dL, 95% CI 3.2, 13.0, *p* = 0.001) driven by a rise in post-load glucose. There was no difference in the mean glucose levels during the OGTT in the first trimester and the postpartum period [66].

### 9.2. OGTT in Early Pregnancy as a Predictor of Subsequent GDM

GCT with a 50 g glucose load predicted cGDM in some early studies [67,68]. In a cohort of 4300 pregnant women from India, the 2 h post 75 gm glucose load value ≥ 140 mg/dL (7.8 mmol/L) in the first trimester, but not after that, correlated with a family history of DM and fetal loss in previous pregnancy [69]. Nakanishi et al. observed that 47% (69/146) of women diagnosed as eGDM by IADPSG criteria reverted to a normal OGTT without any intervention at 24–28 weeks. Moreover, the pregnancy outcome of cGDM was worse than that of eGDM. Interestingly, the majority (59%) of women with eGDM had high FPG, while the primary (73%) contributor to derangement in the OGTT between 24 and 28 weeks was elevated post-prandial glucose values [70]. In a Finnish population-based cohort, a comparison of OGTTs conducted between 12–16 weeks and 24–28 weeks revealed that the application of the late-pregnancy criteria for the diagnosis of eGDM led to its higher prevalence [71]. However, the RCT by Yeral et al. suggested that an OGTT by IADPSG criteria more reliably predicted cGDM than FPG or the two-step method [52].

The “Vitamin D And Lifestyle Intervention for GDM prevention (DALI)” study found that women with eGDM and BMI ≥ 29 kg/m^2^ represented a metabolically distinct group with higher insulin resistance and obesity and a higher risk of elevated blood pressure, triglyceride, free fatty acids, 3-beta-hydroxybutyrate, and heart rate [72].

### 9.3. OGTT in Early Pregnancy as a Predictor of Pregnancy Outcome

An early study from Oman with 564 pregnant women suggested that early and multiple screening with OGTTs increased the detection rate of GDM with an improvement in clinical outcome [73]. A study of 125 singleton pregnancies diagnosed as eGDM (IADPSG criteria) before 16 weeks of gestation revealed that fasting hyperglycemia was associated with congenital malformation. The mean pre-pregnancy BMI of the study group was 29.1 ± 6.5 k/m^2^ [74]. An OGTT conducted between 18 and 20 weeks correlated with an OGTT at 24–28 weeks and was linked to the development of LGA and neonatal hyperinsulinemia [75].

### 9.4. Summary of OGTT as a Diagnostic Test for eGDM

A first-trimester OGTT with IADPSG cut-offs is not an accurate predictor of cGDM. Close to half of the patients with a deranged first-trimester OGTT revert to normoglycemia at 24–28 weeks without any intervention. More false positives with a first-trimester OGTT are driven mainly by a higher number of women with elevated FPG. A deranged OGTT in the first trimester could also be a marker of metabolic dysfunction in obese women. The implications of higher BMI while interpreting deranged first-trimester OGTT results need further investigation. An OGTT performed later toward the end of the second trimester more closely corresponds to cGDM.

## 10. HbA1c for Diagnosis of eGDM

HbA1c is not a conventionally accepted modality for the diagnosis of cGDM. However, it has some advantages over the OGTT, the accepted diagnostic test for cGDM. The OGTT requires 8 h fasting, involves at least three venipunctures, lacks reproducibility, is time consuming, and often poorly tolerated by pregnant women. Unlike the OGTT, HbA1c may be measured any time of the day and has less biological variation, higher reproducibility, and better analytical stability than glucose measurements [76]. Nevertheless, its use for diagnosing cGDM has not yet been recommended by any current guidelines, as several confounding factors make it difficult to interpret HbA1c in the gravid state [77]. As pointed out by Mosca et al., it is also important to have the correct standardization while measuring HbA1c in pregnancy [78]. The HbA1c assessment methodology should be certified by the National Glycohemoglobin Standardization Program and standardized to the Diabetes Control and Complication Trial reference assay [20].

### 10.1. Physiological Changes in Pregnancy Affecting HbA1c

HbA1c is slightly lower in normal pregnancy than in the non-gravid state due to the increased turnover and decreased half-life of red blood cells [79,80]. From early in the first trimester, HbA1c levels fall, reaching a nadir in the early second trimester [81]. Another critical factor influencing HbA1c levels is iron deficiency, prolonging red cell survival and increasing HbA1c levels [82]. Ethnic differences have also been demonstrated to play a role in the association between HbA1c and pregnancy outcomes, further confounding its diagnostic role [83].

### 10.2. Normative HbA1c Values in Pregnancy

There is no consensus on the usual range of HbA1c during pregnancy. The reference intervals in a multicenter study from Italy for women with normoglycemia were 4–5.5% in the gravid state and 4.8–6.2% in the non-gravid state [78]. Nielsen et al. demonstrated that HbA1c is significantly decreased early in pregnancy and reduced even further later on compared to age-matched non-pregnant women. In women with normoglycemia between 14 and 33 weeks’ gestation, the range of HbA1c was 4.5–5.7% in early and 4.4–5.6% in late pregnancy, while in the non-gravid state, it was 4.7–6.3%. However, hemoglobin levels were not accounted for, and anemia could have inadvertently affected the results [84]. O’Connor et al. reported trimester-specific reference intervals for HbA1c in a study of 246 pregnant women without diabetes and with normal hemoglobin levels. The first-trimester range was 4.8–5.5%, and for the next two, it was 4.4–5.4% in both [85].

### 10.3. Predictive Accuracy of HbA1c in Early Pregnancy to Detect Subsequent GDM

HbA1c ≥ 6.5% is used to define the presence of overt DM in pregnancy [6]. However, there are no internationally accepted cut-offs to diagnose eGDM. The levels suggested by different investigators based on the ability to predict cGDM are outlined in Table 4. A lower threshold of 4.8%, as proposed by Benaiges et al., had an improved sensitivity with a negative predictive value of 95% [86]. Lower values of HbA1c could reliably rule out the risk of cGDM and may identify women not requiring an OGTT between 24 and 28 weeks. Because lower thresholds often have poor specificity and PPVs, their role in predicting the risk of cGDM is limited.

Most studies on the predictive accuracy of HbA1c in early pregnancy to detect cGDM have suggested an HbA1c between 5.25 and 6% with positive PPVs ranging from 0.13 to 0.74 [87,88,89,90,91,92,93,94,95,96,97,98]. The wide range of PPV could result from the different OGTT criteria used for diagnosis, ethnic variations, and iron status in pregnancy. Studies have reported a 98–99% specificity with a slightly higher HbA1C cut-off of 5.9%. However, sensitivity was very low at this value of HbA1c [95,98]. At this threshold, low-risk women who would otherwise not be subjected to an OGTT might benefit from earlier diagnosis and interventions. A recent meta-analysis of ten high-quality studies concluded that the risk of developing cGDM increases with an HbA1c of ≥ 5.7%, and values ≥ 6.0% identify almost all women at risk [99].

### 10.4. First-Trimester HbA1c and Pregnancy Outcomes

The association between HbA1c levels and adverse pregnancy outcomes remains indistinct. Few studies analyzing peri-conception and first-trimester HbA1c measurements and pregnancy outcomes are in women with preexisting DM [100]. Some studies have reported an association between an HbA1c of 5.9% and events such as macrosomia, preeclampsia, major congenital anomaly, and shoulder dystocia [98,101,102,103]. Interestingly, Mane et al. reported that early pregnancy HbA1c was a better predictor of pregnancy outcomes than FPG [104]. On the contrary, elevated HbA1C was not indicative of adverse events in a few studies [105,106]. Ethnic differences can also modify the relationship between HbA1c and study end-points, further confounding the diagnostic role of HbA1c [83].

### 10.5. Summary of HbA1c as a Diagnostic Test for eGDM

In early pregnancy, higher values of HbA1c, albeit below the diagnostic threshold for overt DM, correlate with cGDM. Different groups suggested the cut-off values of ≥5.9, ≥5.7, and ≥5.5% based on the ability to predict cGDM later. Lower thresholds have improved sensitivity but worsened specificity. Increasing the cut point to achieve a higher specificity compromised the test’s sensitivity, limiting its usefulness as a screening test. From the available evidence, HbA1C values ≤ 4.8% and ≥5.9% in early pregnancy seem to be reasonable cut-offs to rule out and predict the risk of cGDM. However, a single HbA1c cut-off that can strike a balance in terms of specificity and sensitivity has not been established, and the role of HbA1c in the diagnostic pathway of eGDM requires further validation. An elevated Hba1c is indicative of adverse pregnancy outcomes in some but not all studies. Ethnic differences have also been demonstrated in the association between HbA1c and pregnancy outcomes. While an earlier intervention might minimize the risk, further studies are necessary before being recommended for clinical use.

**Table 4:** Predictive accuracy of HbA1c in early pregnancy to detect subsequent GDM.

Author (Year of Publication/Country) Study Type	No of Participants	HbA1C Threshold	GDM Diagnostic Test	Sensitivity	Specificity	PPV	NPV
Hughes (2014/New Zealand) Prospective [98]	974	5.9	IADPSG	0.18	0.98	0.52	0.92
Fong (2014/USA) Retrospective [18,93]	526	5.7	Carpenter–Coustan	0.27	0.91	0.27	0.91
Amylidi (2015/Switzerland) Prospective [97] *	208	6	IADPSG	0.74	0.51	-	-
Osmundson (2016/USA) Retrospective [92]	2812	5.7	IADPSG	0.29	-	0.13	0.94
Benaiges (2017/Spain) prospective [86]	1158	4.8	Carpenter–Coustan	0.97	-	-	0.95
Wu (2018/China) Prospective [96]	690	5.25	IADPSG	0.36	0.86	0.15	0.95
Hinkle (2018/USA) Prospective [16,91]	2802	5.5	Carpenter–Coustan	0.47	0.79	-	-
Arbib (2019/Israel) Retrospective [90]	142	5.45	Carpenter–Coustan	0.83	0.69	0.53	0.90
Boe (2019/USA) Prospective [89]	2358	5.5	Carpenter–Coustan	0.56	0.77	0.15	0.96
Bozkurt (2020/Austria) Prospective [88]	220	5.7	IADPSG	0.20	0.96	0.74	0.66
Immanuel (2020/Europe) [87]	869	5.7	IADPSG	0.15	0.89	0.51	0.60
Jamieson (2021/Australia) Prospective [94]	396 Aboriginal-129Non-Aboriginal-267	5.6	ADIPS	0.48 0.04	0.93 0.94	0.71 0.28	0.85 0.68
Sun (2021/China) Prospective [95]	744	5.9	IADPSG	0.02	0.99	-	-

* High-risk cohort, results may not be applicable in GDM women without risk factors. ADIPS—Australasian Diabetes in Pregnancy Society, GDM—gestational diabetes mellitus, PPV—positive predictive value, NPV—negative predictive value, IADPSG—International Association of the Diabetes and Pregnancy Study Group.

## 11. Interventional Studies in eGDM

Several studies have explored the effects of intervention on eGDM (Table 5). There is considerable heterogeneity among the trials related to design, study population, time frame for testing, criteria for cGDM, and treatment targets. The results of these studies have been conflicting. Early screening would aim to initiate timely treatment and potentially improve maternal and neonatal outcomes. However, earlier treatment has not always translated into improved results.

### 11.1. Trials Comparing Early Diagnosis and Treatment versus Regular Diagnosis and Treatment

We identified nine studies comparing early screening and intervention vs. regular screening and intervention [45,107,108,109,110,111,112,113,114]. Among the nine studies, only one showed better neonatal composite outcomes with early intervention [108]. Four studies showed no differences in end-points between treated eGDM and cGDM [45,107,109,114]. Four studies reported worse maternal and fetal effects in the eGDM group compared to those of the regular group [110,111,112,113]. Two other studies have compared the treatment outcomes between women with eGDM and preexisting diabetes. Both studies found that results in the eGDM group despite early diagnosis and treatment approximated those seen with preexisting DM [115,116].

In a meta-analysis of 13 cohort studies by Immanuel et al., perinatal mortality (relative risk (RR) 3.58 (1.91, 6.71)), neonatal hypoglycemia (RR 1.61 (1.02, 2.55)), and insulin use (RR 1.71 (1.45, 2.03)) were greater among eGDM women compared to cGDM women, despite treatment. There was no significant difference between eGDM and cGDM in mean birth weight, LGA, or small for gestational age (SGA). However, the quality of evidence of the included studies, as evaluated by the authors, was low to very low [117].

### 11.2. Studies Comparing Early Diagnosis Followed by Early Treatment versus Standard Care

We identified five studies comparing the effects of early intervention versus the regular standard of care in women with eGDM [118,119,120,121,122]. While three studies found similar outcomes between the two groups [119,120,122], more recently, Casson et al. reported a decreased incidence of pre-eclampsia and LGA births after early intervention [118]. In contrast, Simmons et al., in a pilot RCT on 79 women, reported that treatment could be associated with a play-off between reduced LGA but an increased NICU admission, attributed to higher rates of SGA in the early treatment group [121]. Apart from the uncertainty about the benefits of eGDM treatment, this study also raised concerns that early treatment could result in fetal undernutrition. Though the targets were different, most studies point toward similar or worse outcomes with early treatment of eGDM compared to standard care. At present, there is no consensus on what should be the ideal strategy for eGDM. The targets for treatment in early pregnancy may be different from conventional goals to translate into meaningful clinical outcomes. Larger RCTs are warranted to evaluate optimal treatment objectives in eGDM. Results from three large RCTs, the “Treatment of Booking Gestational diabetes Mellitus” (ToBOGM) study [123], “Prediabetes in pregnancy, can early intervention improve outcomes” PINTO study [124], and the “Effect of Early Screening and Intervention for Gestational Diabetes Mellitus on Pregnancy Outcomes” (TESGO) study [125], are awaited.

**Table 5:** Intervention studies in eGDM.

Author (Year of Publication/Country) Study Type	Study Population	GDM Criteria	Timeframe of Testing For eGDM	Comparison	Treatment Targets	Results in Early GDM Intervention Group as Compared to Control Group	Conclusion/Remarks
Hawkins (2008/USA) [113] Retrospective cohort study	GDM < 24 weeks and treated with diet modifications-339	2-step method 1 h 50 g GCT followed by 3 h 100 g OGTT C-C criteria	<24 weeks	Outcomes in diet-treated GDM diagnosed before 24 weeks vs. after 24 weeks	Not defined	eGDM vs cGDM PE treated with MgSO4 ↑ GA ↓ LI= SD= CS= BW= BW > 4000 g ↑ LGA ↑ 5m APGAR < 4 = NICU adm= NH= SB= ND=	The increased rate of LGA and macrosomic infants did not persist after adjustment for demographic characteristics and weight Diet-treated eGDM have a 2-fold increased risk of PE
	GDM > 24 weeks and treated with diet modifications-2257						
Most (2009/USA) [112] Retrospective cohort study	eGDM-98	2-step method 1 h 50 g GCT followed by a 3 h 100 g OGTT C-C criteria	First trimester	Outcomes following interventions in 2 groups	FPG: 60–90 mg/dL (3.3–5 mmol/l) 2 h PP ≤ 120 mg/dL (6.6 mmol/L)	eGDM vs. cGDM CS↑ PIH= SD= PTD= Macrosomia ↑. LGA ↑ APGAR score=	Adverse perinatal outcome significantly higher in eGDM despite early identification and management
	cGDM-242						
Gupta (2014/USA) [116] Retrospective cohort study	3 cohorts	2-step method 1 h 50 g GCT followed by 3 h 100 g OGTT C-C criteria	<24 weeks	Outcomes following interventions in 3 groups	FPG ≤ 95 mg/dL (5.2 mmol/L) 2 h PP ≤ 140 mg/dL (7.7 mmol/L)	eGDM vs. cGDM BW > 4500 g= 5 m APGAR= In eGDM vs. pregestational DM BW > 4500 g ↓ 5 m APGAR=	Outcomes were similar in eGDM and cGDM Preexisting DM more incidence of BW > 4500 g than eGDM
	(1) eGDM (< 24 weeks)-140						
	(2) preexist-ing DM-63						
	(3) cGDM-221						
Alunni (2015/USA) [45] Retrospective cohort	eGDM-175	eGDM: HbA1c of 5.7–6.4% or FPG 92–125 mg/dL (5.1—6.9 mmol/L) cGDM: C-C criteria	≤24 weeks	Early screening and treatment vs. regular screening and treatment	FPG < 90 mg/dL (5 mmol/L) 1 hr PP < 130 mg/dL (7.2 mmol/L)	GA= CS= BW Macrosomia= SGA=	Early vs. standard screening is associated with similar maternal and fetal outcomes
	cGDM-147						
Sweeting (2015/ Australia) [115] Retrospective study	4 cohorts	ADIPS criteria	<24 weeks	Outcomes following interventions in 4 groups	Study period 1991–1997 FPG < 99 mg/dL (5.5 mmol/L) 2 h PP < 120.6 mg/dL (6.7 mmol/L	eGDM vs. cGDM GA ↓ (<12 w, 12–23 w) PTD ↑ (<12 w, 12–23 w) CS ↑ (12–23 w) PIH ↑ (<12 w, 12–12 w) Macrosomia ↑ (<12 w) Hypoglycemia ↑ (12–23 w) NICU adm ↑ (12–23 w) LGA= SGA= SB =	Despite early testing and treatment, eGDM in high-risk women results in poorer pregnancy outcomes. Outcomes for GDM at <12 weeks approximated those seen in preexisting DM
	(1) Preexist-ing DM-65						
	(2) eGDM < 12 weeks-68						
	(3) eGDM 12–23 weeks-1247				Study period 1998–2011 FPG < 93.6 mg/dL (5.2 mmol/L) 1 h PP < 135 mg/dL (7.5 mmol/L)		
	(4) cGDM ≥ 24 weeks-3493						
Boriboonhirunsarn (2016/Thailand) [111] Retrospective cohort study	eGDM-142	2-step method 1 h 50 g GCT followed by 3 h 100 g OGTT C-C criteria	<20 weeks	Outcomes following interventions in 2 groups	2 h PP < 120 mg/dL (6.6 mmol/L)	eGDM vs. cGDM GA= PE= CS= BW for GA= Macrosomia= Neonatal hypoglycemia= NH=	Optimal gestational weight gain and glycemic control were independently associated with LGA and were more frequent in eGDM
	cGDM-142						
Osmundson 2016/USA) [16] Prospective RCT	HbA1c-5.7–6.4% in early pregnancy without preexisting DM	HbA1C 5.7–6.4%	<14 weeks	Early treatment vs. no treatment	FPG < 92 mg/dL (5.1 mmol/L) 1 h PP < 135 mg/dL (7.5 mmol/L)	GA= CS= PIH= LI= Excess maternal weight gain= Infant BW > 4000 g = C-peptide > 90 percentile =	Early treatment did not improve maternal and fetal outcomes
	Early treatment-42						
	Usual care-41						
Haigwara (2018/Japan) [114] Retrospective cohort study	eGDM-528	2 h 75 g OGTT/ IADPSG criteria	<20 weeks	Early screening and treatment vs. usual screening and treatment	HbA1C < 5.8% Glycoalbumin < 15.8%	eGDM vs cGDM PIH↓ BW= Macrosomia= LGA= SGA= SD= CS= NICU adm= Neonatal hypoglycemia= RDS=	Early treatment did not improve fetal outcomes
	cGDM-147						
Bashir (2018/Qatar) [110] Retrospective cohort study	eGDM-273	2 h 75 g OGTT/ IADPSG criteria	<24 weeks	Early screening and treatment vs. usual screening and treatment	FPG ≤ 95 mg/dL (5.3 mmol/L) 1 h PP ≤ 140 mg/dL ( 7.8 mmol/L) 2 h PP ≤ 120 mg/dL (6.7 mmol/L)	eGDM vs cGDM GA ↓ BW ↓ Preterm labor ↑ NICU adm ↑ CS ↑ SGA= PIH= LGA= Polyhydramnios = Macrosomia= SD = Neonatal hypoglycemia= LI = NH=	On multivariate logistic regression analysis, cGDM associated with higher risk of macrosomia and neonatal hypoglycemia
	cGDM-528						
Simmons (2018/ Australia) [121] Prospective RCT	Women with risk factors for GDM screened at <20 weeks	2 h 75 g OGTT/ IADPSG criteria	<20 weeks	eGDM women randomized to immediate clinic referral/ongoing treatment or no treatment	FPG < 95 mg/dL (5.3 mmol/L) 2 h PP < 122 mg/dL (6.8 mmol/L)	Early vs no intervention LGA↓ ↑ NICU adm (largely associated with SGA)	Early treatment may result in a play-off between reducing macrosomia but increasing fetal undernutrition and SGA
	GDM-21						
	Immediate clinic referral-11						
	No intervention-10						
Vinter (2018/ Denmark) [120] Prospective RCT	Hyperglycemia (WHO 2013 criteria) * and BMI 30–45	2 h 75 g OGTT/IADPSG criteria	12–15 weeks	Lifestyle intervention vs. no intervention among women diagnosed with eGDM	Not defined	Early vs standrad care PIH= PE= CS ↑ Gestational Age= SD= Cord-blood C-peptide= PTD= LGA= NICU adm=	Early lifestyle intervention in obese eGDM women not effective in improving maternal and fetal outcomes
	Early lifestyle intervention-36						
	Standard care-54						
Bianchi (2019/Italy) [109] Retrospective cohort	High-risk women (prior GDM or pre-pregnancy BMI ≥ 30 or FPG 100–124.9 mg/dL (5.55—6.94 mmol/L) at first visit	2 h 75 g OGTT IADPSG criteria	16–18 weeks	Early GDM screening and treatment vs. routine screening and treatment	Not defined	Early vs standard care GA= CS= PTD= Macrosomia= SGA= LGA= 5 m APGAR ≤ 8 =	Early vs. standard screening and treatment of GDM in high-risk women is associated with similar short-term maternal-fetal outcomes
	eGDM-145						
	cGDM-145						
Roeder (2019/USA) [119] Prospective RCT	eGDM (HbA1c ≥ 5.7% and/or FPG ≥ 92 mg/dL (5.1 mmol/L))	2 h 75 g OGTT IADPSG criteria	≤15 weeks	Early pregnancy vs. usual treatment of hyperglycemia in eGDM	FPG ≤ 90 mg/dL (5 mmol/L) 1 h PP ≤ 130 mg/dL (7.2 mmol/L)	Early vs late treatment C peptide > 90th percentile= Neonatal fat mass= Neonatal weight for length percentile at birth= Macrosomia= Maternal weight gain= GDM Diagnosis=	Early vs. standard screening and treatment is associated with similar maternal and fetal outcomes
	Early treatment-82						
	Third-trimester treatment-75						
Clarke (2020/Australia) [108] Retrospective cohort	High risk women **	2 h 75 g OGTT IADPSG criteria	<24 weeks	Early GDM screening and treatment vs. routine screening and treatment	FPG < 90 mg/dL (5.0 mmol/L) 2 h PP < 120 mg/dL (6.7 mmol/L)	eGDM vs cGDM Primary CS= PIH= PPH= LI= Newborn composite outcome frequency *** ↓	Reduced neonatal morbidity but similar maternal outcomes from early screening
	eGDM-133						
	cGDM-636						
Harper (2020/USA) [107] Prospective RCT	BMI ≥ 30, without ODIP, history of bariatric surgery or prior CS	2-step method 1 h 50 g GCT followed by a 3 h 100 g OGTT C-C criteria	14–20 weeks	Early GDM screening and treatment vs. routine screening and treatment	FPG <95 mg/dL (5.27 mmol/L) 2 h PP < 120 mg/dL (6.6 mmol/L)	Early vs routine management CS= PIH= PE= SD= Neonatal hypoglycemia= LGA= Macrosomia= NH=	Early vs. standard screening and treatment in obese GDM women is associated with similar maternal and fetal outcomes
	Early screening and treatment-459						
	Routine screening and treatment-462						
Cosson (2021/France) [118] Retrospective Cohort	FPG—92–124.2 mg/dL (5.1–6.9 mmol/L) before 22 weeks	2 h 75 g OGTT IADPSG criteria	<22 weeks	Immediate intervention in women with fasting hyperglycemia vs. no intervention	Not defined	Immediate vs no intervention PE↓ LGA (in subset with FPG ≥ 99 mg/dL (5.5 mmol/L)) ↓	Treating early fasting hyperglycemia, especially when FPG is ≥ 99 mg/dL (5.5 mmol/L) may improve maternal and fetal outcome
	Early intervention-255						
	Retested at or after 22 weeks and treated if GDM-268						

* Modified WHO 2013 criteria: FPG 92 mg/dL (≥5.1 mmol/L), 2 h CBG > 153 mg/dL (8.5 mmol/L) ( 75 g OGTT). ** Previous GDM, age ≥ 40 years, BMI > 35 kg/m^2^ (height and weight measured at booking visit) first-degree family history of diabetes mellitus, previous macrosomia, polycystic ovarian syndrome, corticosteroid or antipsychotic use, and non-Caucasian ethnicity. *** Lower newborn composite outcome—hypoglycemia, birth trauma, NICU/SCN adm, SB, ND, respiratory distress, and phototherapy. ADIPS—Australasian Diabetes in Pregnancy Society, adm—admission, BMI—body mass index, BW—birth weight, C-C—Carpenter and Coustan, CS—Caesarean section, eGDM—early gestational diabetes mellitus, FPG—fasting plasma glucose, GA—gestational age, GDM—gestational diabetes mellitus, GCT—glucose challenge test, IADPSG—International Association of the Diabetes and Pregnancy Study Group, LGA—large for gestational age, LI—labor induction, NICU—neonatal intensive care unit, ND—neonatal death, NH—neonatal hyperbilirubinemia, ODIP—overt diabetes in pregnancy, OGTT—oral glucose tolerance test, PE—pre-eclampsia, PIH—pregnancy-induced hypertension, PP—post-prandial, PTD—preterm delivery, RDS—respiratory distress syndrome, RCT—randomized control trial, SB—stillbirth, SGA—small for gestational age, SD—shoulder dystocia, WHO—World Health Organization, =—similar, ↓—decreased or lower, ↑—increased or higher.

### 11.3. Summary of Intervention Studies

The studies that have explored the effects of early interventions on eGDM have been heterogeneous for the study design, study population, time frame for eGDM testing, criteria for defining cGDM, and treatment targets. Nevertheless, most studies point toward similar or worse outcomes in eGDM than cGDM despite early recognition and treatment. Some studies have even reported results identical to overt DM in treated eGDM. Fetal undernutrition and SGA are other concerns with early treatment. Targets in early pregnancy that could translate into meaningful clinical outcomes without the risk of undernutrition and SGA births require investigation.

## 12. Summary and Recommendations

### 12.1. Fasting Plasma Glucose

Intermediate elevations in FPG in early pregnancy correlate with an increased risk for cGDM and adverse feto-maternal events. There is inadequate evidence, however, to suggest a specific cut-off value for early pregnancy FPG that could predict future GDM. FPG ≥ 92 mg/dL (5.1 mmol/L) in early pregnancy is associated with worsened perinatal outcome, including a higher chance of LGA infants and macrosomia, even in the absence of the later development of cGDM. Pre-pregnancy BMI in the overweight range or above, in conjunction with FPG ≥ 92 mg/dL (5.1 mmol/L), may represent pregnancies at higher risk of complications and should be considered for close monitoring.

Considering the limitations of the currently available literature, and the possible association between fasting hyperglycemia and worsened perinatal outcome, the earlier proposed criteria of FPG ≥ 92 mg/dL (5.1 mmol/L) can be reconsidered as a plausible threshold for the diagnosis of eGDM. The same cut-off will help to maintain consistency and uniformity but needs to be validated in large multicenter studies.

### 12.2. OGTT and HbA1c

A deranged one-step (with IADPSG cut-off) or two-step (Carpenter–Coustan criteria) OGTT in early pregnancy does not predict cGDM consistently and does not have sufficient data to support its usage as a screening test for eGDM. Early pregnancy HbA1c ≥ 5.9% is associated with an increased risk of cGDM and might correlate with worse perinatal outcomes. However, there is insufficient data to recommend it as a screening test for eGDM.

### 12.3. Effect of Intervention

Intervention studies for diagnosing and treating eGDM before the conventional time frame of 24–28 weeks failed to show consistent benefits. Some studies even indicate that early treatment might lead to fetal growth restriction. There is inadequate evidence to recommend the optimal treatment strategy for eGDM, and it might vary from no intervention to lifestyle change to the initiation of insulin. There is also no consensus on the optimal glycemic target for eGDM and whether a strategy similar to cGDM can be applied to manage it. Finally, adopting an early intervention strategy for eGDM might lead to unnecessary medicalization of pregnancies and pose a considerable burden on resources. There is also a possible risk of negative impact on the mental health of the expecting mother and the family. The medical benefits, psychological impact, and logistic feasibility should be taken into consideration while defining the approach.

### 12.4. Pathophysiology

We also propose that the pathophysiology of eGDM and cGDM might be different. The available literature suggests that fasting hyperglycemia is the predominant derangement and the driver behind worsened perinatal outcomes in eGDM. On the contrary, it is well established that postprandial hyperglycemia is predominantly associated with LGA and macrosomia and other adverse effects of cGDM. A closer scrutiny of this possible mechanistic difference merits further investigation and might guide us in framing the optimal approach.

## 13. Conclusions

A considerable number of women are diagnosed as having intermediate levels of hyperglycemia in early pregnancy. There is no consensus on the optimal diagnostic and therapeutic approach for this large group having eGDM. FPG, OGTT, and HbA1c have been explored as diagnostic strategies to detect eGDM. A diagnostic cut-off for these indices have not been identified, but elevated values correlate with the risk of subsequent GDM and adverse feto-maternal events. Emerging data suggests that the association between early pregnancy fasting hyperglycemia and adverse perinatal end-points holds irrespective of the development of late-pregnancy GDM. Higher pre-pregnancy BMI could be an additional determinant of unfavorable outcomes. The intervention studies on the treatment of eGDM demonstrate conflicting results, partially attributed to heterogeneity in study designs. Well-designed, multi-ethnic, multicentric clinical trials to define diagnostic and therapeutic strategies for eGDM, taking into consideration the clinical and economic implications, are needed.

## Figures and Tables

**Table 2:** Screening methodology suggested by different organizations for diagnosing hyperglycemia early in pregnancy.

Organization/ Society (Year)	Test	Criteria	Timing	Target Population	Comments
American Diabetes Association (ADA) (2020) [20]	FPG 2 h PG (75 g OGTT) HbA1c RPG	**Diagnose DM complicating pregnancy if** FPG ≥ 126 mg/dL (7.0 mmol/L) 2 h PG ≥ 200 mg/dL (11.1 mmol/L)	First prenatal visit	Women with risk factors ^a^	Limitation and lack of evidence regarding applicability of IADPSG or 2-step criteria in first half of pregnancy recognized and need for further work noted
		HbA1c ≥ 6.5% (48 mmol/mol)			
		RPG ≥ 200 mg/dL (11.1 mmol/L) with classic symptoms of hyperglycemia			
		Diagnose GDM if lower threshold for GDM met (specifics not defined)			
World Health Organization (2013) [7]	FPG 1 h PG 2 h PG (75 g OGTT)	**Diagnose GDM if** FPG = 92–125 mg/dL (5.1–6.9 mmol/L)	Not defined	Not defined (need to be decided by individual countries)	Definition of GDM applies to any time in pregnancy. Acknowledges that benefit of diagnosing and treating GDM before the usual window of 24–28 weeks’ gestation is not established.
		1 h PG ≥ 180 mg/dL (10.0 mmol/L)			
		2 h PG = 153–199 mg/dL (8.5–11.0 mmol/L)			
	FPG 2 h PG RPG	**Diagnose DM in pregnancy if** FPG ≥ 126 mg/dL (7.0 mmol/L)			
		2 h PG ≥ 200 mg/dL (11.1 mmol/L)			
		RPG ≥ 200 mg/dL (11.1 mmol/L) in the presence of diabetes symptoms			
American College of Obstetricians and Gynecologists (ACOG) (2018) [8]	Best test is not clear. ADA diagnostic criteria for nonpregnant individuals could be used [20].	Standard ADA criteria [20]	Preferably at initiation of prenatal care	Women with risk factors ^b^	HbA1C can be used but may not be suitable for use alone because of decreased sensitivity compared with OGTT
IADPSG (2010) [6]	FPG HbA1c RPG	**Diagnose overt DM** FPG ≥ 126 mg/dL (7.0 mmol/L)	First prenatal visit	Decision to perform test on all pregnant women or only for those at high risk to be made on basis of the background frequency of abnormal glucose metabolism in the population and on local circumstances	
		HbA1c ≥ 6.5% (48 mmol/mol)			
		RPG ≥ 200 mg/dL (11.1 mmol/L) + confirmation by FPG or HbA1c			
		**Diagnose GDM** FPG ≥ 92 mg/dL (5.1 mmol/L) but <126 mg/dL (7.0 mmol/L)			
International Federation of Gynecology and Obstetrics (FIGO) (2015) [16]	WHO criteria for diagnosis of DM and IADPSG criteria for diagnosis of GDM [6,25]	As described	Not defined	All pregnant women should be tested for hyperglycemia during pregnancy by 1-step strategy	In many low-resource countries, alternate strategies should also be considered
Australasian Diabetes in Pregnancy Society (ADIPS) (2014) [17]	**One moderate risk factor ^c^** FPG or RPG followed by pregnancy OGTT (0 h, 1 h, 2 h) if clinically indicated	IADSPG criteria for diagnosis of GDM	Early in pregnancy	Depending on risk factors	Thresholds for further action are not clear at present and clinical judgement should be exercised
	**2 moderate or 1 high risk factor** Pregnancy OGTT	The use of the term “overt diabetes” not recommended	At first opportunity after conception		
National Institute for Health and Care Excellence (NICE) (2015) [15]	**2 h 75 g OGTT** FPG 2 h PG	**Diagnose GDM if** FPG > 100 mg/dL (5.6 mmol/L)	After booking (whether in the first or second trimester)	Risk factor based, to be assessed at the booking appointment. ^d^	Suggests against FPG, RPG, HbA1c, GCT, or urinalysis for glucose to determine risk of developing GDM
		2 h PG > 140 mg/dL (7.8 mmol/L)			

^a^ Risk factors defined by ADA—Overweight or obese (BMI ≥ 25 kg/m^2^ or ≥23 kg/m^2^ in Asian Americans) adults who have one or more of the following risk factors: 1. 1st-degree relative with DM, 2. high-risk race/ethnicity (e.g., African American, Latino, Native American, Asian American, Pacific Islander), 3. history of cardiovascular disease, 4. hypertension (≥140/90 mmHg or on therapy for hypertension), 5. HDL cholesterol level < 35 mg/dL (0.90 mmol/L) and/or a triglyceride level > 250 mg/dL (2.82 mmol/L), 6. women with polycystic ovary syndrome, 7. physical inactivity, 8. other clinical conditions associated with insulin resistance (e.g., severe obesity, acanthosis nigricans). ^b^ Risk factors defined by ACOG—All risk factors defined by ADA with additionally 1. previously given birth to an infant weighing 4000 g (approximately 9 lb) or more, 2. previous GDM, 3. HbA1C ≥ 5.7%, impaired glucose tolerance or impaired fasting glucose on previous testing. ^c^ ADIPS moderate risk factors—1. Ethnicity: Asian, Indian subcontinent, Aboriginal, Torres Strait Islander, Pacific Islander, Maori, Middle Eastern, non-white African, 2. BMI 25–35 kg/m^2^. ADIPS severe risk factors—1. Previous GDM, 2. previously elevated blood glucose level, 3. maternal age ≥ 40 years, 4. family history of DM (1st-degree relative with diabetes or a sister with GDM), 5. BMI > 35 kg/m^2^, 6. previous macrosomia (baby with birth weight > 4500 g or >90th centile), 7. polycystic ovarian syndrome, 8. medications: corticosteroids, antipsychotics. ^d^ NICE risk factors—1. BMI > 30 kg/m^2^, 2. previous baby with macrosomia weighing 4.5 kg or above, 3. previous GDM, 4. family history of DM (first-degree relative with DM), 5. minority ethnic family origin with a high prevalence of DM. GDM—gestational diabetes mellitus, DM—diabetes mellitus, FPG—fasting plasma glucose, PG—plasma glucose, RPG—random plasma glucose, IADPSG—International Association of the Diabetes and Pregnancy Study Group, GCT—glucose challenge test.

## Data Availability

Not applicable.
